# The Great Pretender: Rectal Syphilis Mimic a Cancer

**DOI:** 10.1155/2015/434198

**Published:** 2015-09-15

**Authors:** Andrea Pisani Ceretti, Matteo Virdis, Nirvana Maroni, Monica Arena, Enzo Masci, Alberto Magenta, Enrico Opocher

**Affiliations:** ^1^Department of HBP & Digestive Surgery, University of Milan, San Paolo Hospital, Via A. Di Rudinì 8, 20142 Milan, Italy; ^2^Department of Digestive Endoscopy, San Paolo Hospital, Via A. Di Rudinì 8, 20142 Milan, Italy; ^3^Department of Diagnostic & Interventional Radiology, University of Milan, San Paolo Hospital, Via A. Di Rudinì 8, 20142 Milan, Italy

## Abstract

Rectal syphilis is a rare expression of the widely recognised sexual transmitted disease, also known as the great imitator for its peculiarity of being confused with mild anorectal diseases because of its vague symptoms or believed rectal malignancy, with the concrete risk of overtreatment. We present the case of a male patient with primary rectal syphilis, firstly diagnosed as rectal cancer; the medical, radiological, and endoscopic features are discussed below.

## 1. Introduction

In the last fifteen years, sexual transmitted diseases (STD) had a new increase in incidence; among them syphilis plays a primary role, above all in men who have sex with men (MSM) [1].

Its anorectal localization is particularly rare and constitutes a challenge for the triad surgeon-radiologist-GI endoscopist because it is often misinterpreted as malignant rectal pathology [2].

We present the case of a male patient firstly diagnosed for anorectal cancer and then discovered to be a form of anorectal syphilis.

## 2. Case Report

A 48-year-old HIV-positive white male was admitted in our proctology outpatient clinic due to proctorrhagia arisen a few weeks before. Digital rectal examination revealed a palpable mass extended for 3 cm on the right-anterior wall of the rectum, 5 cm away from the anal verge.

Investigating patient's clinical history and his sexual preferences, we knew he had no relevant medical history, had an ongoing homosexual relationship, and denied recent unsafe sexual behaviour.

An urgent colonoscopy was arranged and confirmed a single large ulcerated lesion, occupying one-third of the visceral circumference; biopsy specimens were sent for histological analysis.

The patient underwent a complete staging in the suspect of rectal malignancy; the computed tomography was negative for metastases to thoracic and abdominal parenchymal organs. The MRI showed a pathological, 2 cm deep thickening of the rectal wall, starting at 4 cm from the anal verge and extended proximally for about 6 cm, with a possible infiltrative behaviour to the mesorectal fat and significant postcontrast signal enhancement but no hyper intensity in the diffusion-weighted images (*b* = 800, 1000); two centimetric lymphadenopathies were also identified in the perirectal fat, a possible expression of nodal metastases. A Positron Emission Tomography reported a focal [F-18]FDG accumulation in the rectal wall, expression of a lesion with high glucose metabolism, and in five spots in the mesorectal space, suggestive for nodal spread.

At last a transanal ultrasound was performed and documented a uT3 level of invasion and an N+ involvement, without involvement of* levator ani* muscle.

No evidence of neoplastic cells was found in bioptic specimen; subsequently the patient underwent two more endoscopic procedures with extensive sampling of the ulcerated lesion, always obtaining the result of a chronic inflammatory process, with a negative research for HSV 1/2, CMV, and HHV-8 antigens.

Only at this point the patient was screened for genitourinary infectious diseases; he was positive for syphilis on serological testing, and Warthin-Starry stain allowed direct visualization of spirochetes on biopsy specimen; thus diagnosis of rectal syphilitic ulcer was made.

The patient was then addressed to the Infectious Diseases Division where he was treated with 2.4 mega units of Benzathine Penicillin with subsequent complete remission of the disease.

## 3. Discussion

In the last decade syphilis had an increasing incidence as in Europe as in the USA.

The major spread occurred between homosexual and bisexual young men, mainly in poor and disadvantaged social groups and in big crowded cities; 25–50% cases of syphilis in MSM present HIV coinfection, which can make healing more difficult and lead to relapse of the disease [3, 4].

For these reasons a complete anamnestic interview, also including sexual behaviour and preferences, has a primary importance in diagnosis.

Infection by* Treponema pallidum* represents the third cause of symptomatic anorectal infection between MSM, following HSV and Gonorrhoea [1].

Primary and secondary anorectal syphilis is, though a rare expression of the systemic disease, reported in literature just through a dozen case reports.

Typically initial clinical presentation includes a single ulcer, called* chancre*, located in the mid-distal portion of rectum or a rubbery mass alone or with satellite lesions with different degree of ulceration;* kissing-like* lesion can also be found specular to the main* chancre*.

It is uncommon to find other colonic portions involved in the disease [5].

Typical symptoms referred by patient include variations in bowel habits, mucorrhea, hematochezia, tenesmus, itching, anal discharge, and defecatory urgency, common to all benign anorectal pathologies and in some cases of malignancy [6].

Differential diagnosis, which can be really challenging for the surgeon, should include Inflammatory Bowel Disease, lymphoma,* lymphogranuloma venereum*, herpetic ulcer and other viral ulcers, solitary rectal ulcer, and rectal cancer [2].

Because of that, even in the case presented above the final diagnosis needed several special consultations.

**Figure 1:**
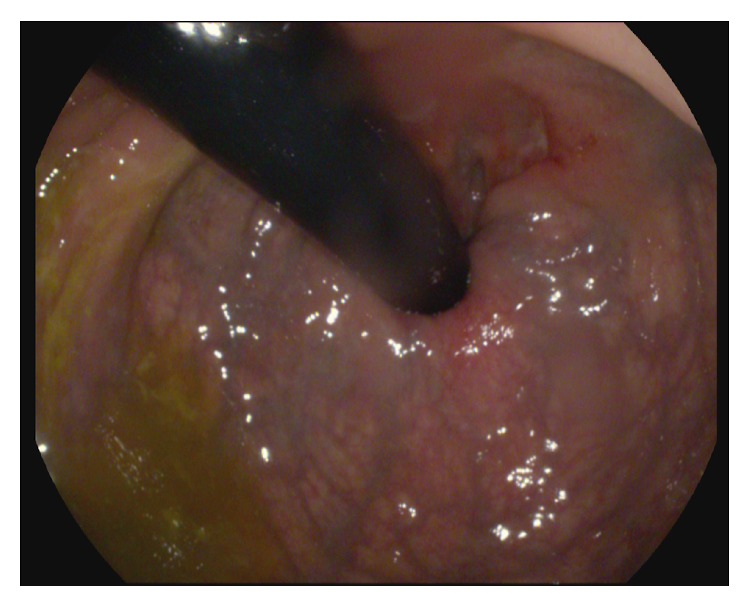
An endoscopic retroversion image of rectal ampulla showing an ulcerated lesion at the posterior commissure.

**Figure 2:**
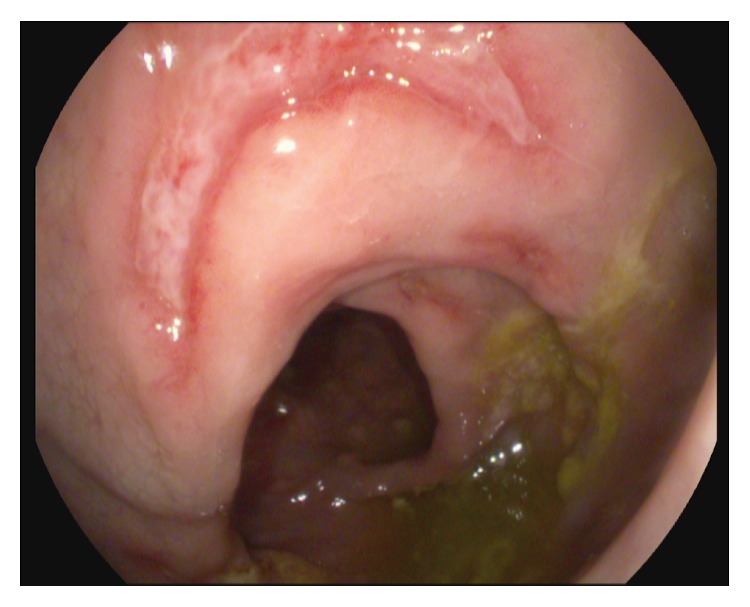
A single ulcer with regular edges and a lunate shape, occupying one-third of the visceral circumference.

The spectrum of endoscopic findings of rectal syphilis is various and includes proctitis, masses, ulcers, and pseudotumors. The most common macroscopic presentation of primary rectal syphilis is a cancer-like mass with varying degrees of erosion and ulceration suggestive for cancer. In this case we observed a single ulcer with regular edges and a lunate shape, occupying one-third of the visceral circumference. The endoscopic appearance that we described was not uniquely indicative of neoplastic ulcer. Therefore, any anorectal ulcer that is not typical of carcinoma or other conditions should be viewed with suspicion. Endoscopist should perform multiple biopsies, because standard histological evaluation may occasionally be nonspecific [7, 8] (Figures 1 and 2).

MRI showed thickness of the rectal walls with homogeneous contrast enhancement after administration of gadolinium, lymph nodes, and oedema of the mesorectal fat, a typical behaviour of rectal cancer, but in diffusion-weighted images performed with different *b* values (800, 1000); there was no hyperintensity of the suspected neoplastic lesion, that is, a classical MRI sign of both rectal adenocarcinoma and mucinous carcinoma [9].

**Figure 3:**
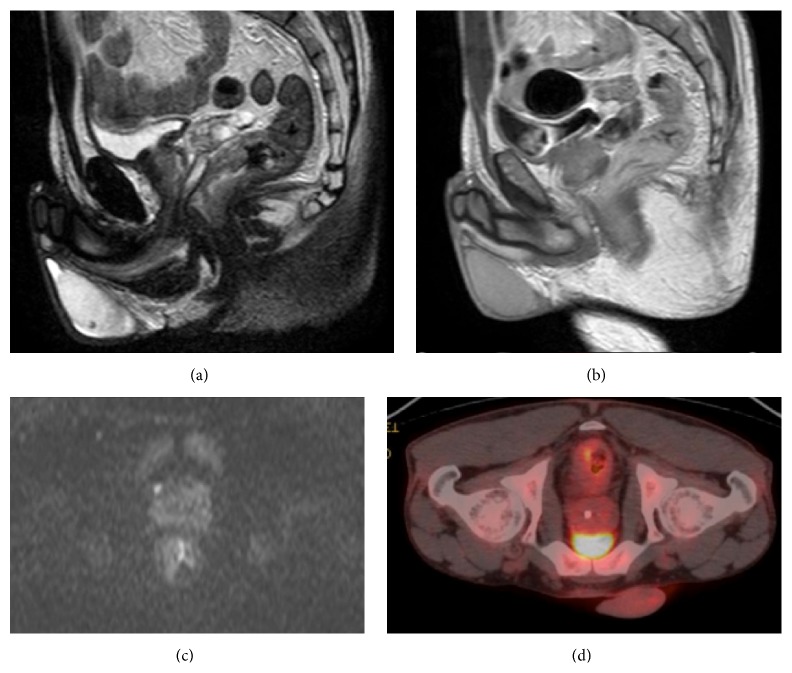
(a) T2 weighted sagittal plan with isointense rectal wall thickness; (b) T1 weighted sagittal plan after gadolinium administration with homogeneous contrast enhancement of the rectal walls; (c) Axial diffusion-weighted image (*b* = 1000) with no hyper intensity of the rectal wall; (d) 18F FDG PET-CT with focal FDG accumulation in the rectal wall.

Performing a 18F-FDG PET-CT does not solve the problem because a focal [F-18]FDG accumulation in the rectal wall and nodes in mesorectal fat is common to lesions with high glucose metabolism [10, 11] (Figure 3).

Histological examination and dark field microscopy on fresh specimen, together with a positive serological test like VDRL, are fundamental for the definitive diagnosis.

Haematoxylin-Eosin stain shows chronic aspecific inflammation concerning tissues, vessels, and perivascular space, with presence of plasma-cells and giant multinucleated cells; Warthin-Starry stain allows direct visualization of spirochetes in bioptic specimen [12].

Once the diagnosis has been clarified, the therapy consists of 2.4 million units of intramuscular Penicillin G-Benzathine a week for a minimum of three weeks. Regression of lesions and infectious disease is usually rapid and complete, without residual manifestations [7].

In conclusion the incidence and prevalence of syphilis and in particular its anorectal form rose significantly in the last 15 years alone or associated with other STD or HIV, mainly between men who have sex with men. It is also called the great imitator because of the wide range of proctologic diseases that must be excluded in order to have a precise diagnosis, firstly rectal neoplasm.

For this reason we believe that endoscopist, surgeon, and radiologist should deeply investigate patient's sexual habits and routinely perform STD screening to avoid unnecessary diagnostic procedures and overtreatments and to provide the anatomopathologist the most complete picture of the patient in order to obtain the best answer.

## Abbreviations

MSM: Male who has sex with male
HIV: Human immunodeficiency virus
FDG: Fluorodesossiglucose
HSV: Herpes simplex virus
CMV: Cytomegalovirus
HHV: Human herpes virus
PET-CT: Positron emission tomography-computed tomography
STD: Sexual transmitted disease.
